# Anti-Inflammatory Property of *Plantago major* Leaf Extract Reduces the Inflammatory Reaction in Experimental Acetaminophen-Induced Liver Injury

**DOI:** 10.1155/2015/347861

**Published:** 2015-08-02

**Authors:** Farida Hussan, Adila Sofea Mansor, Siti Nazihahasma Hassan, Tg. Nurul Tasnim Tengku Nor Effendy Kamaruddin, Siti Balkis Budin, Faizah Othman

**Affiliations:** ^1^Department of Anatomy, Faculty of Medicine, Universiti Kebangsaan Malaysia, Jalan Yaacob Latif, Bandar Tun Razak, Cheras, 56000 Kuala Lumpur, Malaysia; ^2^Department of Biomedical Science, Faculty of Health Sciences, Universiti Kebangsaan Malaysia, Jalan Raja Muda Abdul Aziz, 50300 Kuala Lumpur, Malaysia

## Abstract

Hepatic injury induces inflammatory process and cell necrosis.* Plantago major* is traditionally used for various diseases. This study aimed to determine the anti-inflammatory property of* P. major* leaf extracts on inflammatory reaction following acetaminophen (APAP) hepatotoxicity. Thirty male Sprague-Dawley rats were divided into 5 groups, namely, normal control (C), APAP, aqueous (APAP + AQ), methanol (APAP + MT), and ethanol (APAP + ET) extract treated groups. All APAP groups received oral APAP (2 g/kg) at day 0. Then, 1000 mg/kg dose of* P. major* extracts was given for six days. The levels of liver transaminases were measured at day 1 and day 7 after APAP induction. At day 7, the blood and liver tissue were collected to determine plasma cytokines and tissue 11*β*-HSD type 1 enzyme. The in vitro anti-inflammatory activities of methanol, ethanol, and aqueous extracts were 26.74 ± 1.6%, 21.69 ± 2.81%, and 12.23 ± 3.15%, respectively. The ALT and AST levels were significantly higher in the APAP groups at day 1 whereas the enzyme levels of all groups showed no significant difference at day 7. The extracts treatment significantly reduced the proinflammatory cytokine levels and significantly increased the 11*β*-HSD type 1 enzyme activity (*p* < 0.05). In conclusion, the* P. major* extracts attenuate the inflammatory reaction following APAP-induced liver injury.

## 1. Introduction

Inflammation is a body homeostasis in response to any type of tissue injury. It is a complex phenomenon which involves innate and adaptive immune responses. It initiates leucocytes migration, compliment system stimulation, macrophages activation, and cytokines production from activated macrophages and neutrophils. Cytokines such as tumour necrosis factor-*α* (TNF-*α*) and interleukins (IL) play important roles in recruitment of neutrophils and activation of macrophages to accelerate tissue repair process.

Drug-induced liver injury causes acute liver failure [1]. Acetaminophen (APAP) is one of the drugs which induce liver damage [2]. APAP-induced liver injury is due to its toxic metabolites N-acetyl-p-benzoquinone imine (NAPQI). Excessive production of NAPQI in APAP toxicity leads to glutathione (GSH) depletion, resulting in binding of the NAPQI to cellular protein which triggers cell injury [3]. The liver injury develops within three to five hours following exposure of APAP toxic dose and reaches the peak at 12 hours [4]. Cell injury elicits inflammatory reaction in the liver [5].

11*β*-Hydroxysteroid dehydrogenase type 1 (11*β*-HSD type 1) enzyme is a native enzyme in liver and interconverts inactive glucocorticoids (cortisone) to active cortisol which has anti-inflammatory action [6]. Anti-inflammatory action of cortisol suppresses cellular immunity and potentiating of humoral immunity [7]. Therefore, the activity and expression of 11*β*-HSD type 1 enzyme might involve in inflammatory reaction following hepatocytes injury.

*Plantago major* (*P. major*) of Plantaginaceae family is commonly known as broadleaf plantain [8] and it is popular in traditional medicine for wound healing as well as treating diseases related to skin, respiratory organs, digestive organs, reproduction, circulation, cancer, infection, and pain [9]. Besides the traditional applications, many researches have been done to prove its medicinal properties such as antiulcerogenic, anti-inflammatory, and immune-modulating activities and antioxidant, antiviral, and anticarcinogenic activities [10]. In Malaysia,* P. major* has been used as a diuretic, tonic, and cough mixture [11] and to treat urinary calculus [12] and diabetes [13].* P. major* leaves possess numerous bioactive compounds such as flavonoids, terpenoids, pectin, iridoid glycosides, and tannins which express anti-inflammatory and antioxidant activities [8–10]. The present study was aimed at determining the effect of* P. major* on the changes in plasma cytokines such as TNF-*α*, IL-1, IL-6, and IL-10 following APAP-induced liver injury. Furthermore, the changes of 11*β*-HSD type 1 enzyme in liver tissue would be determined and the role of this enzyme against inflammatory reaction in response to liver injury would be discussed.

## 2. Materials and Methods

### 2.1. Experimental Animals

Thirty male Sprague-Dawley rats (200–250 g) were obtained from the institutional animal resource unit. The rats were reared in stainless steel cages with a room temperature of 27 ± 2°C with 12 hours light and dark cycle. All rats were allowed to access food and tap water* ad libitum*. All the animal handling procedures were in accordance with the ethical guideline with the approval number UKMAEC: FP/ANAT/2013/FARIDA/25-SEPT./533-OCT.-2013-SEPT.-2014.

### 2.2. Extract Preparation

The* P. major* plant is collected from Cameron Highland, Malaysia. The leaves were plucked and cleaned with tab water. Air-dried ground leaves powder of* P. major* (100 g for each solvent) was macerated in three different solvents such as 60% methanol, 60% ethanol, and deionized water, respectively. The solutions were kept for 3 days in a dark room at 22 ± 3°C. The mixture was then filtered and the supernatant was collected. The process was repeated three times. The three batches of supernatant were mixed. The aqueous extract was freeze-dried whereas the methanol and ethanol extracts were concentrated in rotary evaporator at 50–60°C. Then, the concentrated fluid was later freeze-dried into powder. The extract powder was then sent to the Forest Research Institute of Malaysia (FRIM) to evaluate anti-inflammatory activity with lipoxygenase assays.

### 2.3. Lipoxygenase Assay

This assay was done according to the method developed by Malik et al. [14] with slight modification. Soybean lipoxygenase (1.13.11.12) type I-B and linoleic acid were purchased from Sigma (St. Louis, MO, USA). In assay protocol, 160 *μ*L of 100 mM sodium phosphate buffer (pH 8.0), 10 *μ*L of test-compound solution, and 20 *μ*L of lipoxidase enzyme solution were mixed and incubated for 10 min at 25°C. The reaction was then initiated by the addition of 10 *μ*L linoleic acid (substrate) solution, with the formation of (9Z, 11E)-(13S)-13-hydroperoxyoctadeca-9,11-dienoate and the change in absorbance was measured at 234 nm. Test compounds and the positive control were dissolved in DMSO. All the reactions were performed in triplicate in 96-well microplate in Tecan Infinite M200 Microplate Reader (Tecan, Austria). The IC50 values were then calculated using the GraphPad Prism Analysis.

### 2.4. Experimental Design

The rats were acclimatised for one week prior to administering any test agents and then divided into two groups. Normal control group (C, *n* = 6) received oral 0.9% normal saline (NS) throughout the experiment whereas APAP group (*n* = 24) which was subdivided into APAP, APAP + AQ, APAP + MT, and APAP + ET received oral APAP (2 g/kg). The subgroups received 0.9% NS and aqueous, methanol, and ethanol extracts, respectively, for 6 days. The extract dose (1000 mg/kg) was chosen based on our preliminary study. At day 1 which was 24 hours after the APAP induction, the blood was collected from the retroorbital space to determine the plasma liver enzyme. At day 7, the rats were anaesthetised and the blood was collected via cardiac puncture. Then, the rats were sacrificed and the liver was harvested.

### 2.5. Biochemical Analysis

The collected blood was put in EDTA tube and centrifuged at 1000 ×g in 4°C for 10 minutes. The plasma was collected and stored at −80°C until further analysis. Liver enzyme levels at days 1 and 7 were determined by semiautomatic method, using Bioanalyzer, semiautomatic BTS-350, BioSystems S.A., Spain. The plasma cytokine levels at day 7 were tested using ProcartaPlex cytokine assay kits (eBioscience, USA). Liver tissue was rinsed with phosphate buffer saline (PBS) and prepared for tissue homogenisation and immunostaining. The activity of 11*β*-HSD type 1 enzyme was determined in liver tissue using enzyme-linked immunosorbent assay (ELISA) kit (Uscn, USA).

### 2.6. Immunohistochemical Staining

The formalin fixed liver tissue was incubated in pH 6 citrate buffers at 95°C for 30 minutes and 0.3% hydrogen peroxidase for 10 minutes at room temperature. The primary antibody used was rMR64-82-2D6 and anti-rabbit IgG was used as a secondary antibody. The presence of 11*β*-HSD type 1 enzyme was detected using the streptavidin-peroxidase system and 3,3′-diaminobenzidine (DAB). Lastly, it was counterstained with Harris haematoxylin.

### 2.7. Statistical Analysis

The data was presented as mean ± standard error of mean (SEM). The normally distributed data were analyzed using parametric analysis of variance (ANOVA) test. The data that were not normally distributed were analyzed using nonparametric tests, Mann-Whitney *U* test. The significant value was set as *p* < 0.05. All mentioned statistical analyses were conducted using Statistical Product and Service Solutions (SPSS) software, version 13.

## 3. Results

The lipoxygenase inhibition of the extracts was found to be 26.74 ± 1.6%, 21.69 ± 2.81%, and 12.23 ± 3.15% in the methanol, ethanol, and aqueous extracts, respectively. The results were presented as mean ± SEM (% inhibition). The standard, nonselective lipoxygenase inhibitor, nordihydroguaiaretic acid (NDGA), showed 99.86 ± 0.14% inhibition at the final concentration of 100 *μ*g/mL. The methanol and ethanol extract showed higher antilipoxygenase activity than the aqueous extract.

### 3.1. Changes in Liver Enzymes

The liver enzymes such as alanine aminotransferase (ALT) and aspartate aminotransferase (AST) were significantly increased in APAP groups compared to the control group at day 1 which was 24 hours after the APAP (2 g/kg) induction. The liver enzyme levels in all the APAP groups at day 7 were significantly reduced compared to day 1 (*p* < 0.05). At day 7, there was no significant change of AST and ALT levels in all groups except the aqueous extract treated group which revealed significant lower ALT level than the APAP group of the same day (*p* < 0.05). The results were shown in Table 1.

### 3.2. Changes of Inflammatory Cytokines

**Figure 1:**
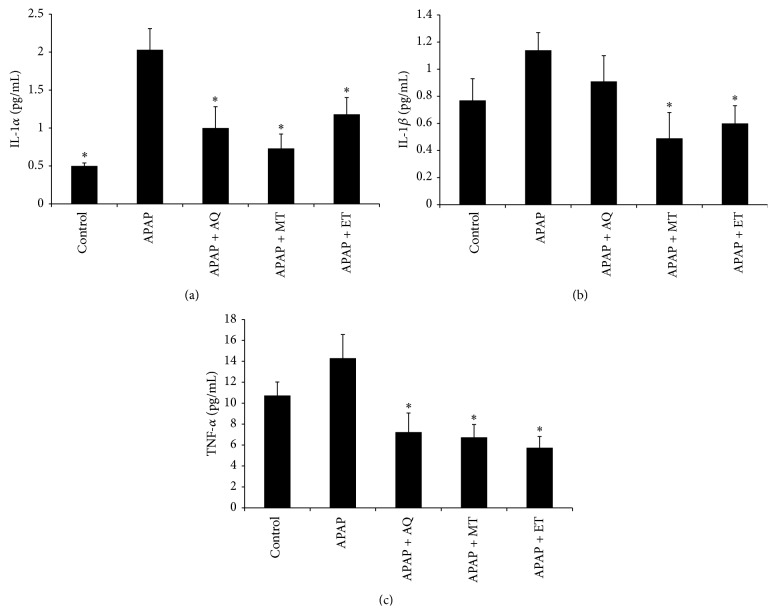
Effects of three different* P. major* extracts on the level of proinflammatory cytokines in APAP-induced rats: (a) IL-1*α*, (b) IL-1*β*, and (c) TNF-*α*.  ^*∗*^Significant difference from the APAP group (*p* < 0.05).

**Figure 2:**
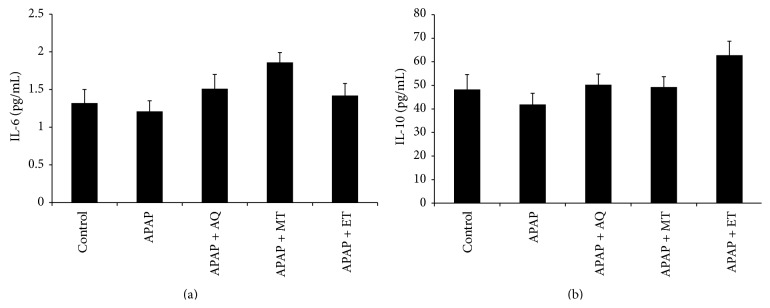
Effects of three different* P. major* extracts on the level of anti-inflammatory cytokines in APAP-induced rats: (a) IL-6 and (b) IL-10 (no significant difference *p* > 0.05).

The mean levels of IL-1*α*, IL-1*β*, and TNF-*α* in all groups at day 7 were shown in Figure 1. The IL-1*α* level in the APAP group was significantly higher than that of the control group (*p* < 0.05) (Figure 1(a)). However, there was no significant difference in IL-1*β* and TNF-*α* between the control and the APAP group (Figures 1(b) and 1(c)). The level of IL-1*α*, IL-1*β*, and TNF-*α* in the APAP group was significantly higher compared to the treated groups (*p* < 0.05) which indicated that* P. major* treatment significantly reduced the proinflammatory cytokines. The mean levels of IL-6 and IL-10 in all groups were shown in Figure 2. The levels of IL-6 and IL-10 in all groups showed no significant difference (*p* > 0.05).

### 3.3. Changes of 11*β*-HSD Type 1 Enzyme Activity

**Figure 3:**
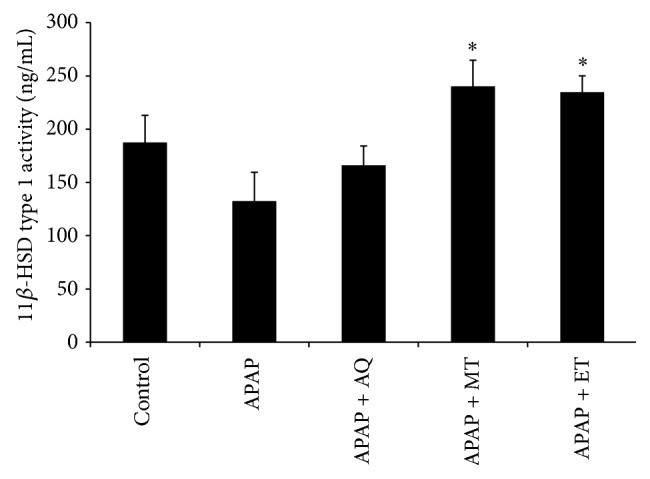
Effects of three different* P. major* extracts on the activity of 11*β*-HSD type 1 enzyme in APAP-induced liver tissue;  ^*∗*^significant difference from the APAP group (*p* < 0.05).

The activity of 11*β*-HSD type 1 enzyme in all groups was shown in Figure 3. The enzyme activity between the control and APAP groups showed no significant difference whereas the activity was significantly reduced in APAP group compared to the APAP + MT and APAP + ET groups (*p* < 0.05). Therefore, the methanol and ethanol extract were able to enhance the enzyme activity following APAP-induced liver injury.

### 3.4. 11*β*-HSD Type 1 Enzyme Expression

**Figure 4:**
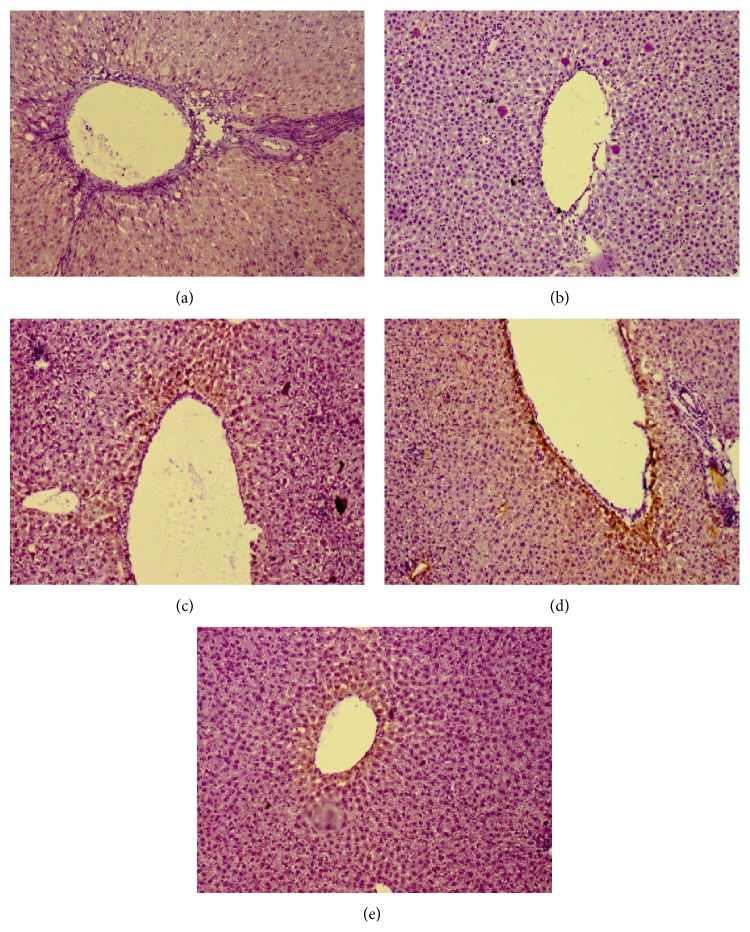
11*β*-HSD type 1 enzyme expression in liver tissue (×10; immunohistochemical stain): (a) control; (b) APAP; (c) APAP + AQ; (d) APAP + MT; and (e) APAP + ET.

Figure 4 showed the expression of 11*β*-HSD type 1 enzyme in liver tissue. The immunohistochemical staining of 11*β*-HSD type 1 enzyme was used to determine the expression of enzyme. There was no expression of 11*β*-HSD type 1 in the APAP group (Figure 4(b)). However, the greater intensity of expression was found around the central vein area in both APAP + MT and APAP + ET groups (Figures 4(d) and 4(e)).

## 4. Discussion

This study was aimed at determining the effect of* P. major* extract treatment on inflammatory reaction following APAP toxicity. The APAP dose in the present study was proven to produce liver toxicity in rats which showed increased liver enzyme levels and liver necrosis [15]. The treatment dose of 1000 mg/kg of* P. major* extract was based on our preliminary results. The use of high dose (2000 mg/kg) of* P. major* showed no toxic effect [16]. The minimum effective dose of this plant is 600 mg/kg [13].

The* in vivo* anti-inflammatory activity of* P. major* has been well described [17]. The anti-inflammatory activity of the extracts was determined using lipoxygenase assay. Lipoxygenase enzyme catalyses arachidonic acid to produce leukotrienes. Leukotrienes play role in inflammatory diseases. The plant extracts or the phytochemicals which possess inhibitory effect on this enzyme have the potential to be used in inflammatory condition [18]. In the present study, the highest anti-inflammatory activity was found in the methanol extract followed by the ethanol extract. The result was similar to the finding of Beara et al. [19]. It is stated that its anti-inflammatory activity is contributed by flavonoids such as baicalein and hispidulin and iridoid glycosides such as aucubin [9].

Inflammation is a body response to remove tissue debris and to initiate tissue regeneration following injury. Persistence inflammatory reaction exaggerates tissue damage, resulting in improper tissue repair process. Inflammatory reaction is initiated by the release of proinflammatory cytokines such as IL-1, IL-6, and TNF-*α* [20]. The proinflammatory cytokine in the circulation is a signal to recruit neutrophils and leucocytes which subsequently remove the cellular debris to promote tissue regeneration [21]. IL-6 also acts as an anti-inflammatory cytokine by inhibiting the secretion of TNF-*α* and IL-1 [22]. Extensive tissue damage can be prevented by the action of anti-inflammatory cytokines including IL-10 or suppression of proinflammatory cytokines.

In this present study, the proinflammatory cytokine (IL-1*α*, IL-1*β*, and TNF-*α*) levels were significantly high in the APAP group compared to the treated groups. The IL-1 and TNF-*α* which are released by activated macrophages stimulate leukocyte adhesion to endothelial surfaces prior to migration into tissues. IL-1 often expresses synergistic action with TNF-*α* to initiate cell death [23]. The TNF-*α* and IL-1 levels in the plasma reflect the severity of inflammation [24].

The inhibition of TNF-*α* and IL-1 exhibits partial prevention of APAP toxicity, including reduction of liver enzyme in circulation [25]. The results of the present study showed the lower level of TNF-*α* and IL-1 in the* P. major* treated groups. The effect was more pronounced in the methanol extract treated group which was in line with the* in vitro* anti-inflammatory activities of* P. major*. It indicated that the anti-inflammatory property of the* P. major* was able to prevent the inflammatory reaction following APAP toxicity. It has been documented that the natural products with anti-inflammatory properties decreased the levels of proinflammatory cytokines [26].

11*β*-HSD type 1 is present in liver and its role is to interconvert inactive glucocorticoid to the active form [27, 28]. Cortisol in human or corticosterone in rodent is an active form of glucocorticoid which has anti-inflammatory properties. Glucocorticoids express its anti- inflammatory action by suppressing the nuclear transcription factors AP-1 and NF-*κ*B which induce genes expression of all proinflammatory cytokines [29]. In the present study, the* P. major* extract treated groups showed increased activity and expression of 11*β*-HSD type 1 enzyme especially in the methanol and ethanol extract treated groups. Therefore, attenuation of inflammatory reaction might be related to the local tissue production of active glucocorticoid. Furthermore, Dinarello [29] stated that glucocorticoid increases the transcription of anti-inflammatory proteins such as IL-10 and the IL-1 type 2-decoy receptor. However, in the present study, the anti-inflammatory cytokine (such as IL-6 and IL-10) levels were not changed significantly among all groups. Based on our results, the glucocorticoid action activated by 11*β*-HSD type 1 has potential role in suppression of proinflammatory molecules, rather than promoting anti-inflammatory cytokines.

## 5. Conclusion

In conclusion, the leaf extracts of* P. major* have the potential in attenuation of the inflammatory response by reducing the levels of proinflammatory cytokines. The potential mechanism of reduction in inflammatory reaction by the* P. major* extracts could be due to production of local tissue glucocorticoid. Therefore, methanol and ethanol extracts of* P. major* have potential to be used as an alternative or adjunct treatment to reduce inflammation-mediated cell injury following APAP toxicity.

## Figures and Tables

**Table 1:** Effects of three different *P. major* leaf extracts on aspartate aminotransferase (AST) and alanine aminotransferase (ALT) enzymes at day 1 and day 7 after APAP induction.

Groups	AST at day 1 U/L	AST at day 7 U/L	ALT at day 1 U/L	ALT at day 7 U/L
Control	109.67 ± 1.74	133.50 ± 1.44	52.17 ± 1.41	70.00 ± 0.67
APAP	223.97 ± 3.36^a^	145.33 ± 1.73^c^	109.58 ± 1.78^a^	77.58 ± 0.43^c^
APAP + AQ	254.11 ± 3.80^a^	137.25 ± 1.97^c^	94.58 ± 1.20^a^	55.33 ± 0.58^b^
APAP + MT	219.33 ± 2.98^a^	158.00 ± 1.06^c^	117.75 ± 1.39^a^	74.92 ± 0.63^c^
APAP + ET	174.17 ± 3.37^a^	106.33 ± 1.16^c^	103.08 ± 1.3^a^	74.42 ± 0.82^c^

^a^Significant difference from the control group of the same day (*p* < 0.05).

^b^Significant difference from the APAP group of the same day (*p* < 0.05).

^c^Significant difference between the same group of day 1 and day 7 (*p* < 0.05).
